# Supplementary material for: Transcriptomic and Proteomic Analyses of Myzus persicae Carrying Brassica Yellows Virus
Source: Biology (Basel). 2023 Jun 25;12(7):908. doi: 10.3390/biology12070908 (PMC10376434; doi:10.3390/biology12070908)
Supplement: Supplementary file 1 [file biology-12-00908-s001.zip › Figure S1 Confirmation of the presence of BrYV in viruliferous and non-viruliferous aphids by RT-PCR..pdf]

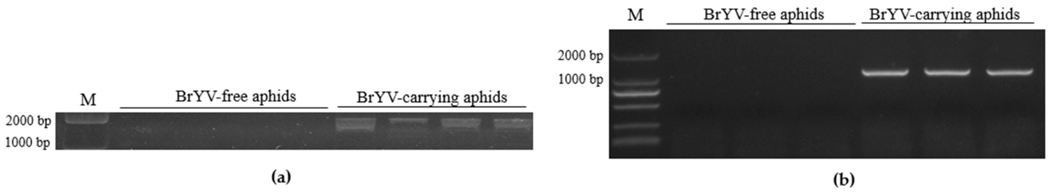

Supplementary Figure S1. Confirmation of the presence of BrYV in viruliferous and non-viruliferous aphids by RT-PCR.

M: DNA ladder. (a) Identification of the samples for RNA sequencing by RT-PCR using universal primers of poleroviruses (PoconF: GAYTGYTCYGGTTTTGACTGG / PocoCPR: CGTCTACCTATTTSGGRTTN). (b) Identification of the samples for TMT analysis by RT-PCR using BrYV-specific primers (Br2674F: GATTGTTCTGGTTTTGACTGG /Br3778R: TTATACTCATGGTAGGCCTTGAG).
