# Supplementary material for: Transcriptomic and Proteomic Analyses of Myzus persicae Carrying Brassica Yellows Virus
Source: Biology (Basel). 2023 Jun 25;12(7):908. doi: 10.3390/biology12070908 (PMC10376434; doi:10.3390/biology12070908)
Supplement: Supplementary file 1 [file biology-12-00908-s001.zip › Table S1 Primers used for qRT-PCR validation.pdf]

Table S1. Primers used for RT-qPCR validation.

Omics	Gene name	Sequence
Transcriptomics / Proteomics	qPCR-actin	F：CGGTTCAAAAACCCAAACCAG<br>R：TGGTGATGATTCCGTGTTC
Transcriptomics	alpha-2 chain-like	F: TGGATCTGGGATGGGTTCAAGG<br>R: TGATGAGTGGCCAAGGCAGT
	probable isocitrate dehydrogenase (NAD) subunit alpha	F: CCTCATCTCCCACCGACGAC<br>R: TCAGGACCGATTCCATCGCC
	trifunctional enzyme subunit alpha	F: TTGGATTCCCGGTTGGTGCT<br>R: AGCCACTGTCCACCATGCTT
	cytochrome c oxidase subunit 5B	F: CCTACCTCCGTACCCGTCCT<br>R: AATTTCGCGGAAGAGGCAGC
	aldose 1-epimerase-like	F: TGTGGGATGGTTATGTGGCAGA<br>R: ACCGAAATGTTGTCCGGGCT
	beta-galactosidase-like	F: TGGATTTCGGTGCCTCGACT<br>R: ACTGGTGATGGTTCACGCCA
	uncharacterized LOC111032711	F: TGGTTCGGTCGGTGCTGATT<br>R: GGGTAATGCCCTTGCAGCAC
	cuticle protein 65-like	F: AGTGTTGATCGCCTTCGTGC<br>R: GGCACGTTTGTCCTTGACGG
	general odorant-binding protein 28a	F: GTGGCGTGTCTGTGCTTGTC<br>R: CGGAACCCTTTGAGTGCGTC
	cuticle protein 21-like	F: CGCCTACCCAGCACCTTCAT<br>R: CGATGACTGACAGGTGGCCT
Proteomics	phosphatidylinositol 4-kinase beta	F: CCTTAGCCTTGACTTTCGGTCA<br>R: CAGGACACTCAGGACTTGGTA
	programmed cell death protein 2	F: AGTGAACCAGCTGAACCCCA<br>R: TGGACTTGCACCCGCATACTTTA
	zinc finger martin-type protein 2	F: GCGAAAATGGGACCGAGACG<br>R: GACGCGGTAATCTCGTTGCT
	protein EFR3-like	F: CTTCGGTTCCACCGGTCTCA<br>R: GCCGCCGTACGGTCAATAAT
	serine/threonine-protein kinase SMG1	F: TGGATCAACTGGGAGGCAGC<br>R: CGCCCACATTTTTATTCCACCTTC
	uncharacterized protein LOC111034038	F: AGGATGCTGAATTGGCTGGGA<br>R: TCCATGGCACATCTCCTACTGA
	baculoviral IAP repeat-containing protein 5-like	F: AGGAAATGGCCGAAGCAGGT<br>R: TCTTCCCAAGGCTTGTCGGT
	xylulose kinase	F: TTGGAGACACGATCAGCCGT<br>R: GTGACGACGGCGACCAACTA
	transmembrane protein 65	F: TCAAAGTTTGGACGCCCCAG<br>R: GGGTTCTGGTACAGTTTCCGTATT
	otoferlin-like	F: GACCGAGCTCTTCCGATCACA

	R: GGTGACTCTGGCCGAGAACT
