# Supplementary material for: Transcriptomic and Proteomic Analyses of Myzus persicae Carrying Brassica Yellows Virus
Source: Biology (Basel). 2023 Jun 25;12(7):908. doi: 10.3390/biology12070908 (PMC10376434; doi:10.3390/biology12070908)
Supplement: Supplementary file 1 [file biology-12-00908-s001.zip › Table S2 Q20 and Q30 values for M. persicae transcriptomes..pdf]

Table S2. Q20 and Q30 values for *M. persicae* transcriptomes.

Samples	Q20	Q30
BrYV-free 1	97.64	93.36
BrYV-free 2	97.73	93.52
BrYV-free 3	97.59	93.19
BrYV-carrying 1	97.73	93.48
BrYV-carrying 2	97.12	92.29
BrYV-carrying 3	97.33	92.70
