# Supplementary material for: Transcriptomic and Proteomic Analyses of Myzus persicae Carrying Brassica Yellows Virus
Source: Biology (Basel). 2023 Jun 25;12(7):908. doi: 10.3390/biology12070908 (PMC10376434; doi:10.3390/biology12070908)
Supplement: Supplementary file 1 [file biology-12-00908-s001.zip › Table S3 Subcellular localization of DEPs.pdf]

Table S3. Subcellular location of DEPs.

Subcellular Location	Protein Number	Protein ID
Nuclear	10	XP_022176845.1; XP_022166015.1; XP_022175607.1; XP_022176292.1; XP_022180011.1; XP_022172089.1; XP_022163038.1; XP_022175975.1; XP_022168101.1; XP_022170749.1
Plasma Membrane	5	XP_022174752.1; XP_022173196.1; XP_022177587.1; XP_022162222.1; XP_022166101.1
Extracellular	2	XP_022179818.1; XP_022183363.1
Cytoplasmic	2	XP_022177587.1; XP_022180011.1
Lysosomal	1	XP_022166101.1
