# Supplementary material for: Transcriptomic and Proteomic Analyses of Myzus persicae Carrying Brassica Yellows Virus
Source: Biology (Basel). 2023 Jun 25;12(7):908. doi: 10.3390/biology12070908 (PMC10376434; doi:10.3390/biology12070908)
Supplement: Supplementary file 1 [file biology-12-00908-s001.zip › Table S4 Log2 Ratio of selected DEGs compared with the result of RT-qPCR verification..pdf]

**Table S4.** $Log_2$ Ratio of selected DEGs compared with the result of RT-qPCR verification.

| Omics | NCBI Reference Sequence | Name | $Log_2$ Ratio | RT-qPCR verification |
|---|---|---|---|---|
| Transcriptomics | XM_022327351.1 | *M. persicae* tubulin alpha-2 chain-like | 4.76 | +[a] |
| | XM_022309470.1 | Probable isocitrate dehydrogenase (NAD) subunit alpha | 1.64 | + |
| | XM_022319592.1 | trifunctional enzyme subunit alpha | 1.01 | + |
| | XM_022316251.1 | cytochrome c oxidase subunit 5B | 1.33 | + |
| | XM_022320945.1 | aldose 1-epimerase-like | 3.20 | + |
| | XM_022319034.1 | beta-galactosidase-like | 1.25 | -[b] |
| | XM_022316814.1 | cuticle protein 65-like | 6.56 | + |
| | XM_022309323.1 | general odorant-binding protein 28a | 3.12 | + |
| | XR_002604334.1 | uncharacterized LOC111032711 | -1.50 | + |
| | XM_022322335.1 | cuticle protein 21-like | 2.64 | - |

[a]"+" indicates the expression levels of the selected DEGs verified by RT-qPCR were consistent with the results of transcriptomics. [b]"-" indicates the expression levels of the selected DEGs verified by RT-qPCR were inconsistent with the results of transcriptomics.
