# Supplementary material for: Transcriptomic and Proteomic Analyses of Myzus persicae Carrying Brassica Yellows Virus
Source: Biology (Basel). 2023 Jun 25;12(7):908. doi: 10.3390/biology12070908 (PMC10376434; doi:10.3390/biology12070908)
Supplement: Supplementary file 1 [file biology-12-00908-s001.zip › Table S5 Ratio of selected DEPs compared with the result of RT-qPCR verification..pdf]

Table S5. Ratio of selected DEPs compared with the result of RT-qPCR verification.

Omics	NCBI Reference Sequence	Name	Ratio	RT-qPCR verification
Proteomics	XP_022160991.1	phosphatidylinositol 4-kinase beta	1.47	+ [a]
	XP_022166015.1	programmed cell death protein 2	1.21	+
	XP_022172089.1	zinc finger matrin-type protein 2	1.27	+
	XP_022176292.1	protein EFR3-like	1.23	+
	XP_022176845.1	serine/threonine-protein kinase SMG1	1.25	+
	XP_022170749.1	uncharacterized protein LOC111034038	1.23	- [b]
	XP_022175607.1	baculoviral IAP repeat-containing protein 5-like	1.22	+
	XP_022179818.1	xylulose kinase	1.21	+
	XP_022177587.1	transmembrane protein 65	0.82	-
	XP_022168101.1	otoferlin-like	0.81	+

[a]"+" indicates the expression levels of the selected DEPs verified by RT-qPCR were consistent with the results of proteomics. [b]"-" indicates the expression levels of the selected DEPs verified by RT-qPCR were inconsistent with the results of proteomics.
